# Expanding the role of *HsfA9* in cold adaptation: the bermudagrass *CdHsfA9* confers cold tolerance in *Arabidopsis* via a novel regulatory module

**DOI:** 10.3389/fpls.2025.1731555

**Published:** 2025-12-19

**Authors:** Lu Gan, Jiayu Wang, Xuebing Yan

**Affiliations:** College of Animal Science and Technology, Yangzhou University, Yangzhou, China

**Keywords:** bermudagrass, cold stress, *D6PKL*, Hsf, IAA

## Abstract

Low-temperature stress limits the growth and geographical distribution of bermudagrass (*Cynodon dactylon* (L.) Pers.), an important warm-season grass worldwide. While heat shock transcription factors (Hsfs) are well known for modulating heat stress responses, their functions in cold adaption remain poorly characterized. In this study, we identified and functionally characterized *CdHsfA9*, a novel Hsf gene isolated from bermudagrass, which localizes to the nucleus. Phylogenetic analysis revealed that *CdHsfA9* clusters closely with *HsfA9* orthologs from cereal plants and *Arabidopsis AtHsfA8*. Expression profiling indicated rapid upregulation under cold stress, with transcript abundance higher in leaves than in roots. Heterologous overexpression of *CdHsfA9* in *Arabidopsis* promoted IAA content and enhanced cold tolerance, as evidenced by increased chlorophyll content, reduced leaf discoloration, and MDA accumulation in transgenic lines compared to mutants and wild-type plants under cold conditions. Transcription factor - centered yeast one-hybrid (TF-Y1H) assay identified 39 motifs bound by CdHsfA9, with target genes enriched in the metabolic and signal transduction pathways. Among these targets was serine/threonine-protein kinase D6PK-like, a key regulator in phosphorylation-related signaling. This direct binding of CdHsfA9 to the *D6PKL* promoter was confirmed by dual luciferase reporter assays in tobacco and ChIP-qPCR in *Arabidopsis*. Our findings extend the functional scope of *HsfA9* beyond heat stress to include low-temperature adaptation and elucidate a broader stress regulatory network involving the *CdHsfA9*-*HSEmotif*-*D6PKL* module and its crosstalk with auxin. This study provides valuable genetic resources and theoretical foundations for improving cold resistance in forage grasses via molecular breeding.

## 1 Introduction

Temperature is a critical environmental factor governing plant growth and development. Exposure to extreme or prolonged abnormal temperatures severely impacts plant survival and productivity (Hasanuzzaman et al., 2013). To mitigate temperature stress, plants have developed intricate regulatory mechanisms, with transcription factors playing a crucial role in sensing stress signals and modulating the expression of stress-responsive genes (Ding et al., 2020).

Heat shock transcription factors (Hsfs) are particularly important, as they recognize heat shock elements (HSEs) in target promoters, thereby regulating transcriptional programs essential for stress adaptation (Andrási et al., 2021; Kharisma et al., 2022). In *Arabidopsis*, HsfA1d and HsfA1e bind to HSE motifs in the *HsfA2* promoter to activate its expression, thereby conferring thermotolerance (Nishizawa-Yokoi et al., 2011); they also interact with the *Hsp101* promoter to regulate basal thermotolerance (Tiwari et al., 2020). These findings highlight the importance of elucidating the Hsf-mediated regulatory network to identify key downstream targets as a basis for understanding diverse stress responses. The Hsf family is structurally conserved yet functionally diverse. Plant Hsfs are categorized into A, B, and C classes based on the length of oligomerization domain (Jiang et al., 2021). Comparative genomics have demonstrated species-specific expansion and diversification within this family (Rehman et al., 2021; Wang et al., 2018; Zafar et al., 2016). For instance, *Arabidopsis* possesses 21 *Hsf* genes (Guo et al., 2008), while soybean has 52 members (Li et al., 2014). In monocots, such as rice, Brachypodium, and foxtail millet, contain 25 or more Hsf loci (Mishra et al., 2020; Mittal et al., 2009; Wen et al., 2017). Despite broad conservation between dicots and monocots, certain Hsf subfamilies exhibit significant evolutionary divergence (Scharf et al., 2012). Although HsfA9 is well-established as a key regulator of seed development in dicots (Wang et al., 2024; Zinsmeister et al., 2020), could its functional role in monocots also be related to seed development or thermotolerance?

Expression and functional studies further highlight context specificity across species and stress modalities. Transcript profiling of Hsf transcripts display stress- and species-dependent regulation. For instance, *HsfA4d* is upregulated by heat in rice (Mittal et al., 2009) but downregulated in wheat (Zhou et al., 2019). *HsfB2b* is strongly induced by cold in Zoysiagrass yet repressed in rice (Guan et al., 2019; Wei et al., 2015). Although expression patterns alone do not establish function, they inform hypotheses and network modeling. Functionally, Hsfs contribute not only to thermotolerance but also to tolerance of cold, drought, and salinity. For example, overexpression of *HbHsfA1* and *HbHsfB1* enhanced cold tolerance in rubber tree (Deng et al., 2018). *Vitis amurensis HsfC1* increases thermotolerance and low-temperature tolerance but reduces salt tolerance when overexpressed in *Arabidopsis* (Jiao et al., 2022). Nevertheless, compared with heat stress, Hsf functions in cold tolerance and the underlying mechanisms remain less well resolved. HsfA9 exhibits notable functional divergence between monocots and dicots, but its role in regulating cold tolerance is not fully understood. In rice, *Os03g12370* (annotated as ‘HsfA9-like’) is induced by both cold and heat stress (Chauhan et al., 2011). Phylogenetic analysis often groups monocot *HsfA9* genes, including the putative bermudagrass *CdHsfA9*, within the HsfA8 subfamily, distinct from dicot *AtHsfA9* (Duan et al., 2019; Giesguth et al., 2015). To date, studies in monocots have largely emphasized inducible expression and physiological responses, leaving the molecular mechanisms by which HsfA9-like factors promote low-temperature tolerance in grasses largely unresolved.

Bermudagrass (*Cynodon dactylon* (L.) Pers.), a widely distributed warm-season grass, is important in ecological conservation and lawn systems but is highly susceptible to low temperature. Our previous transcriptome analysis of 48 wild bermudagrass accessions across latitudes revealed that *CdHsfA9* is significantly induced by 5°C treatment in most accessions but not by high temperature (Chen et al., 2021). Building on this observation, the present study aim to (i) define the contribution of *CdHsfA9* to plant cold tolerance, (ii) identify and validate its direct transcriptional targets and associated pathways, and (iii) elucidate the molecular mechanism by which *CdHsfA9* coordinates low-temperature responses. These efforts will resolve a CdHsfA9-centered regulatory network and provide gene resources and a mechanistic foundation for breeding cold-resilient bermudagrass.

## 2 Materials and methods

### 2.1 Plant materials, growth conditions, and cold treatment

Seeds of bermudagrass (cv. ‘common’) were sown in pot filled with soil: sand: perlite (1:1:1, v/v/v) and maintained in a growth chamber at 30/25°C (day/night) with a light intensity of 400 µmol m^-2^ s^-1^ and 60% relative humidity under a 12-h photoperiod. To analyze gene expression across developmental stages and tissues, samples were collected as follows: seedlings were harvested at 20 days post-germination; mature leaves (the second fully expanded leaf from the top) and roots were collected during the vigorous growth stage (approximately 10 weeks after sowing); and spica were sampled at the heading stage (emergence of the inflorescence). For cold stress, plants were exposed to 5°C for 7 d and leaves were sampled at 0, 3, 6, 12, 24, and 48 h for gene expression analysis.

### 2.2 Gene isolation, phylogenetic and domain analyses, and subcellular localization

Total RNA was extracted from bermudagrass leaves, and first-strand cDNA synthesized for *CdHsfA9* open reading frame (ORF) amplification with primers *CdHsfA9*-F and *CdHsfA9*-R (**Supplementary Table 1**) designed from the assembled sequence and genome of bermudagrass (Zhang et al., 2022). The amplicon was sequenced to confirm identity, and the *CdHsfA9* sequence was deposited in GenBank (MG257788).

CdHsfA9 homologs sequence were collected using the BLASTp program and were aligned using the ClustalW program with standard parameters. A phylogenetic tree was constructed in MEGA5 using the neighbor-joining method with 1,000 bootstrap replicates (Sohpal et al., 2010). Conserved domains were analyzed by quering the deduced amino acid sequences against the NCBI Conserved Domain Database (CDD).

For subcellular localization, the *CdHsfA9* coding sequence was fused in-frame to enhanced green fluorescent protein (eGFP) in the *pEZR_K_LN* vector. Rice protoplast isolation, purification, and PEG-mediated transfection were performed as described previously (Datta and Datta, 1999). DNA concentrations in the PEG solution were adjusted to 10 µg/mL, and cells were incubated for 8–10 min at room temperature. Fluorescence signals were observed with a FV1000 confocal laser-scanning microscope (Olympus, Tokyo, Japan). DAPI staining was used to visualize nuclei.

### 2.3 Transcription factor - centered yeast one-hybrid assay and motif analysis

The full-length *CdHsfA9* coding sequence was cloned into pGADT7 vector for expression in yeast strain Y187. It was found that no yeast colonies grew on the defective plate of SD/-Trp/-Leu/-His (SD-TLH) medium supplemented with 50 mM 3-amino-1,2,4-triazole (3-AT). Based on the self-activation test (**Supplementary Figure 1**), 50 mM 3AT was therefore used for subsequent screening. A motif library plasmid was transferred into Y187 containing the pGADT7-*CdHsfA9* plasmid, and transformants were selected on SD-TLH + 50 mM 3-AT. Positive yeast clones were identified by DNA sequencing, and CdHsfA9-interacting motifs were compiled.

All experimentally derived motifs and standard HSE were converted into the format of position-weight matrices (PWMs). The heatmap of motif similarity clustering was drawn based on the pairwise comparison of these 39 motifs and the HSE using the Pearson Correlation Coefficient (PCC) method (Gupta et al., 2007). All experimental motifs were scanned across the promoter set with FIMO to obtain the matched genes for KEGG enrichment analysis. Furthermore, experimentally derived motifs were compared against JASPAR and PlantTFDB database using TOMTOM tool (Bailey et al., 2015; Gupta et al., 2007) to identify matches with known transcription factor (TF), specifically targeting HSF families. For genome-wide target prediction, the PWMs of candidate motifs and HSE were used to scan the 2.0 kb upstream promoter regions of the bermudagrass genome via FIMO (Grant et al., 2011). The predicted binding sites were then cross-referenced with canonical HSE motif. Genes containing promoter regions where the candidate motif binding sites spatially overlapped with HSE and annotated genes mapped to enriched pathways were designated as putative direct targets.

### 2.4 Dual-luciferase transient expression assay

Transient expression in *Nicotiana benthamiana* (5-leaf stage) was performed by *Agrobacterium*-mediated transient transformation. The *CdHsfA9* coding sequence and 620-bp promoter fragment containing the target motif-34 were cloned into *pBinGFP2* (effector) and *pGreenII_0800_LUC* (reporter), respectively (*KpnI*/*BamHI* restriction sites). Agrobacterial suspensions carrying effector and reporter were infiltrated into leaves with a needle-less syringe. After injection, the plants were cultured for 24–48 h prior to imaging on a ChemiDoc system, and exposure settings were optimized for luminescence visualization. The injected leaves were extracted and tested for the activity of firefly luciferase (Fluc) reporter gene in TECAN infinite M200 PRO (Tecan, Mannedorf, Switzerland) according to the instructions of double-luciferase reporter assay kit (Transgen Biotechnology, Beijing, China). The Renilla luciferase (Rluc) gene, driven by the *CaMV 35S* promoter in the *pGreenII_0800_LUC* vector, was used as an internal control to normalize transformation efficiency. The relative luciferase activity (Fluc/Rluc) was calculated and compared between the experimental group (*pBinGFP2-CdHsfA9* + *pGreenII_0800_LUC-motif-34*) and negative control groups (including *pBinGFP2 + pGreenII_0800_LUC-motif-34*, *pBinGFP2-CdHsfA9* + *pGreenII_0800_LUC*, and the double empty vectors).

### 2.5 Genetic transformation and analysis of transgenic *Arabidopsis*

The *CdHsfA9* ORF (*BamHI*/*SpeI*) was inserted into a *pCAMBIA1301* where the original 35S promoter was replaced by the Ubiquitin (*Ubi*) promoter, with a C-terminal FLAG tag. The destination plasmid in *Agrobacterium tumefaciens* strain GV3101 was transformed into *Arabidopsis* by floral dip. The positive transgenic lines of *Arabidopsis* were selected through hygromycin resistance and PCR confirmation.

*Arabidopsis* ecotype Columbia was used as the wild type (WT). The *hsfA8* (AT1G67970) T-DNA insertion mutant SALK_070573C was obtained from the Arabidopsis Biological Resource Center, with homozygous mutants confirmed by PCR. Surface-sterilized T1/T2 seeds were germinated on Murashige and Skoog (MS) medium containing 100 mg/L hygromycin at 25/20°C (day/night) under a 12-h photoperiod. Homozygous T3, WT, and *hsfA8* seeds were germinated on MS medium and transferred to pots containing peat: vermiculite (3:1, v/v) for maturation, cold-response assays, and gene-expression analysis.

To evaluate physiological responses to cold stress, transgenic plants of *CdHsfA9* overexpression (OE) and empty-vector (EV), WT, and *hsfA8* were exposed to 5°C for 7 d. The chlorophyll content of transgenic plants was extracted by soaking 50 mg of fresh leaves in 10 mL dimethyl sulfoxide in the dark for 72 h. Absorbance at 663.2 nm, 646.8 nm, and 470 nm was recorded, and chlorophyll content was calculated according to Hiscox and Israelstam (1979) and Lichtenthaler and Buschmann (2001). The extracted supernatant from the powder of transgenic leaves (about 80 mg) was collected for protein quality, enzyme assays, and malondialdehyde (MDA) content. Lipid peroxidation was estimated by measuring MDA content with some modifications (Dhindsa et al., 1981). Protein concentration was quantified using the Bradford method (Bradford, 1976). The activities of antioxidant enzymes, including superoxide dismutase (SOD), peroxidase (POD), catalase (CAT), and ascorbate peroxidase (APX), were determined according to the method of Zhang and Kirkham (1996).

To further verify the binding of CdHsfA9 to the target motif, leaves from OE lines were cross-linked, and chromatin was isolated and sheared by sonication. Anti-FLAG antibodies were used for immunoprecipitation; input chromatin served as control. Eluted DNA was purified and analyzed by qPCR to assess enrichment at candidate promoter regions.

### 2.6 RNA extraction and quantitative real-time PCR

Total RNA from bermudagrass or *Arabidopsis* was extracted with TRIzol reagent (Invitrogen, USA). First-strand cDNA was synthesized using PrimeScript RT reagent Kit with gDNA Eraser (Perfect RealTime) (TransGen, China). Quantitative real-time PCR (qRT-PCR) was performed in a total volume of 10 μL containing 1 μL cDNA, 0.5 μL of each primer and 5 μL of SYBR Green mix (TransGen, China) on a Roche LightCycler 96 Sequence Detection System (Roche, USA), with reaction for 5 min at 95°C followed by 40 amplification cycles of 30 s at 95°C, 30 s at 55°C, and 30 s at 72°C. Data were normalized according to the *AtActin2* for *Arabidopsis* (NM_112764.4) or *CdActin* for bermudagrass (Chen et al., 2021) gene expression level and determined by 2^-ΔΔCT^ calculation methods. Primers for qRT-PCR are listed in **Supplementary Table 1**. The analysis included three biological replicates and three technical replicates for each sample.

### 2.7 Statistical analyses

Gene expression levels and cold stress effects and variations among wild type, mutants and transgenic plants for physiological parameters were statistically analyzed using the one-way analysis of variance (ANOVA) followed by Fisher’s LSD test using GraphPad Prism 9. Differences were considered statistically significant at *p* < 0.05.

## 3 Results

### 3.1 *CdHsfA9* encodes an HsfA-family factor localized to the nucleus

**Figure 1:**
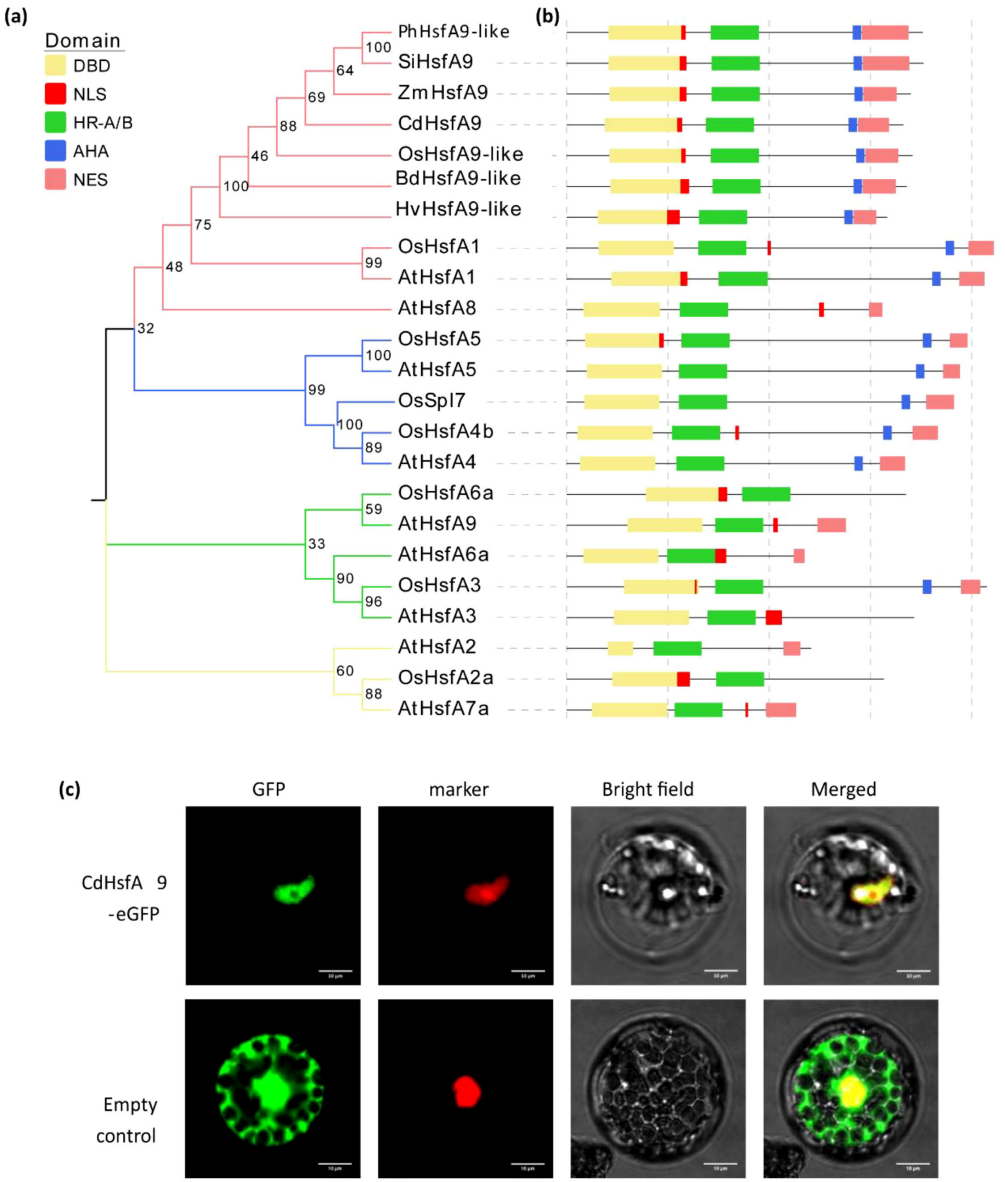
Identification of CdHsfA9. **(a)** Phylogenetic relationships between CdHsfA9 and 22 orthologs constructed in MEGA5 using the neighbor-joining method. At, *Arabidopsis thaliana*; Bd, *Brachypodium distachyon*; Hv, *Hordeum vulgare*; Os, *Oryza sativa*; Ph, *Panicum hallii*; Si, *Setaria italica*; Zm, *Zea mays*. **(b)** Conserved domain architecture of CdHsfA9 and its closest orthologs. DBD, DNA-binding domain; NLS, nuclear localization signal; HR-A/B region, oligomerization domain; AHA motif, activator motif; NES, nuclear export signal. **(c)** Subcellular localization of CdHsfA9. CdHsfA9-GFP fusion in rice protoplasts. Bar = 10 µm.

Based on the assembled *HsfA9* sequence from bermudagrass genome and transcriptome databases, a 1200-bp ORF sequence was amplified from the cDNA library of cultivar ‘common’. BLAST and phylogenetic analysis placed *CdHsfA9* within the Hsf-A group (**Supplementary Figure 2**). Given the functional diversification within HsfAs and the ambigous classification of HsfA8 and HsfA9 between dicots and monocots, further analysis of evolutionary relationship was performed including orthologs from rice, *Brachypodium*, barley, *Arabidopsis*, and etc. (**Figure 1a**). CdHsfA9 showed the highest similarity to HsfA9 orthologs in *Panicum hallii*, *Setaria italica*, and *Zea mays*, and clustered on the same branch as *Arabidopsis* HsfA8 (AtHsfA8).

Conserved domain analysis of the CdHsfA9 polypeptide revealed an N-terminal DNA-binding domain (DBD), an oligomerization domain (HR-A/B), an activator motif (AHA), and a C-terminal nuclear export signal (NES) (**Figure 1b**). A predicted nuclear localization signal (NLS) located between the DBD and HR-A/B suggested nuclear targeting of CdHsfA9. To verify subcellular localization, a CdHsfA9-GFP fusion was transiently expressed in rice protoplasts. The overlap of GFP and DAPI fluorescence indicated nuclear localization of CdHsfA9 (**Figure 1c**).

### 3.2 Expression profile of *CdHsfA9*

**Figure 2:**
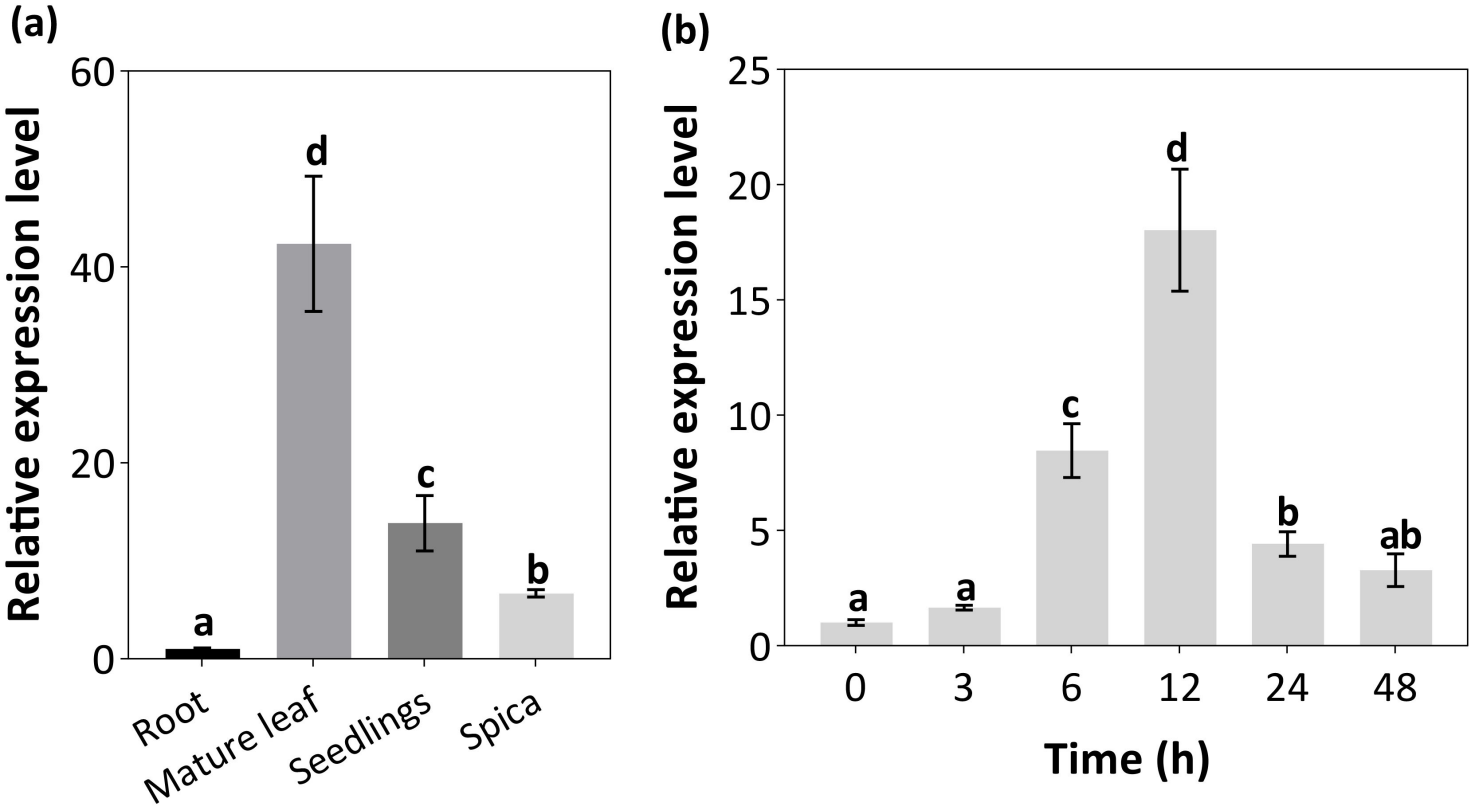
Expression analysis of *CdHsfA9* in bermudagrass. **(a)** Spatio and developmental expression across seedlings (young leaves at 20 days post-germination), mature leaves (the second fully expanded leaf from the top during the vigorous growth stage), and spica (heading stage of the inflorescence emergence). **(b)** Time-series expression under 5°C for 48 h. Relative expression was calculated by the 2^−ΔΔCt^ method with root as the reference. Data are mean ± SD (n = 3 biological replicates). Bars labeled with different letters differ significantly (P < 0.05, Fisher’s LSD).

To assess spatial and development expression of *CdHsfA9*, multiple tissues across bermudagrass growth stage were examined. *CdHsfA9* transcripts were accumulated predominantly in mature leaves, followed by seedlings, spica, and roots (**Figure 2a**). To evaluate cold responsiveness, plants were exposed to 5°C and the relative expression level of *CdHsfA9* were determined in the leaves collected 0, 3, 6, 12, 24, and 48 h after treatment. The transcription levels increased and peaked by 2.5- to 5-fold within 12 h (**Figure 2b**), followed by a gradual decline with prolonged cold exposure (**Figure 2b**).

### 3.3 *CdHsfA9* conferring cold tolerance in *Arabidopsis*

To test the role of *CdHsfA9* in regulating cold tolerance, a FLAG-tagged *CdHsfA9* was expressed constitutively in *Arabidopsis*. Given that CdHsfA9 clustered with AtHsfA8 (**Figure 1a**), the *hsfA8* mutant (SALK_070573C) was included for comparison.

**Figure 3:**
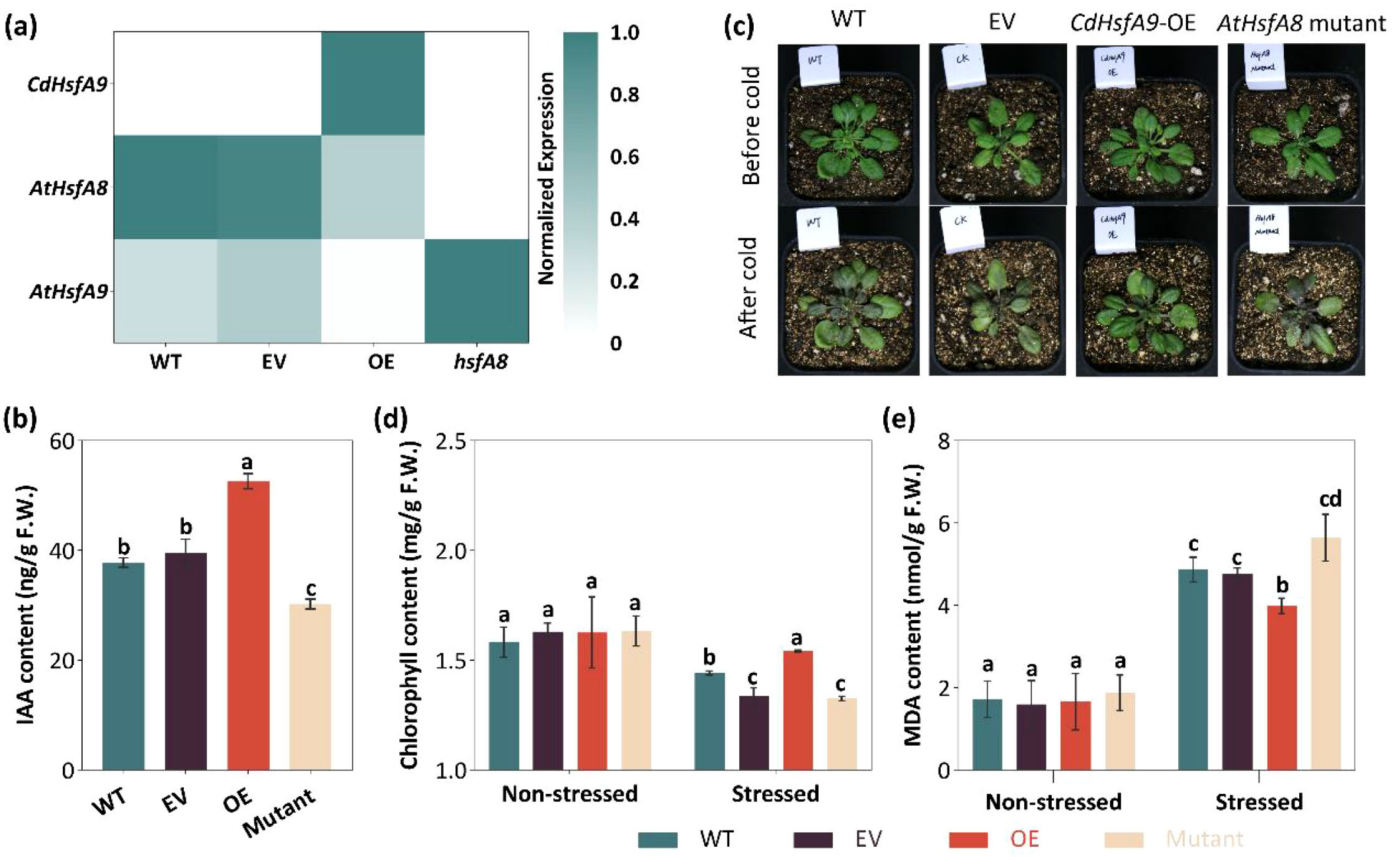
Ectopic expression of *CdHsfA9* in *Arabidopsis*. **(a)** Transcript levels of *CdHsfA9*, *AtHsfA8*, and *AtHsfA9* expression levels in wild type (WT), empty-vector lines (EV), overexpression lines (OE), and the *hsfA8* mutant. The heatmap displays the relative expression levels calculated by the 2^−ΔΔCT^ method. For visualization, values were row-normalized to a scale of 0 to 1. **(b)** IAA content in WT, EV, OE, and mutant plants under non-stress conditions. **(c)** Representative phenotype of 30-day-old plants before cold treatment and after 7 d at 5 °C. **(d)** Chlorophyll content under non-stress and stress condition. **(e)** Malondialdehyde (MDA) content in leaves under non-stress and cold stress conditions. Data are mean ± SD (n = 3 biological replicates). Different letters indicate significant differences (P < 0.05, Fisher’s LSD).

The transcripts of *CdHsfA9* were detected in overexpression lines (OE) but not in wild type (WT), empty-vector lines (EV), or the *hsfA8* mutant, indicating successful transformation (**Figure 3a**). Compared with WT and EV, *AtHsfA8* gene expression was strongly reduced in the mutant, confirming the mutant genotype. Notably, *AtHsfA8* gene expression was also reduced in OE plants, possibly reflecting functional overlap between *CdHsfA9* and *AtHsfA8*. By contrast, *AtHsfA9* expression levels were relatively low in WT and EV lines, they appeared further suppressed in OE plants. Most notably, the *AtHsfA9* transcipt level was significantly upregulated in the *hsfA8* mutant (**Figure 3a**), suggesting functional redundancy and compensation between *AtHsfA9* and *AtHsfA8*. Moreover, IAA content was elevated in OE lines (**Figure 3b**).

Following 7 d at 5°C treatment, the cold stress caused extensive leaf purple/yellow discoloration in WT, EV, and *HsfA8* mutant plants (**Figures 3c, d**). While EV and mutant lines showed similar chlorophyll reduction under cold conditions, analysis of MDA content confirmed that EV and WT suffered comparable levels of cellular damage, distinct from the OE lines (**Figures 3d, e**). In contrast, OE plants exhibited robust cold tolerance, characterized by reduced discoloration, higher chlorophyll retention, and lower MDA accumulation compared to all control groups (**Figures 3c-e**). Consistent with this, OE plants maintained higher antioxidative activities of SOD and POD (**Supplementary Figure 3**). These data collectively demonstrate that heterologous overexpression of *CdHsfA9* enhances cold resistance in *Arabidopsis* by preserving photosynthetic capacity and membrane integrity.

### 3.4 CdHsfA9 directly targets a D6PK-like kinase via an HSE-like motif

**Figure 4:**
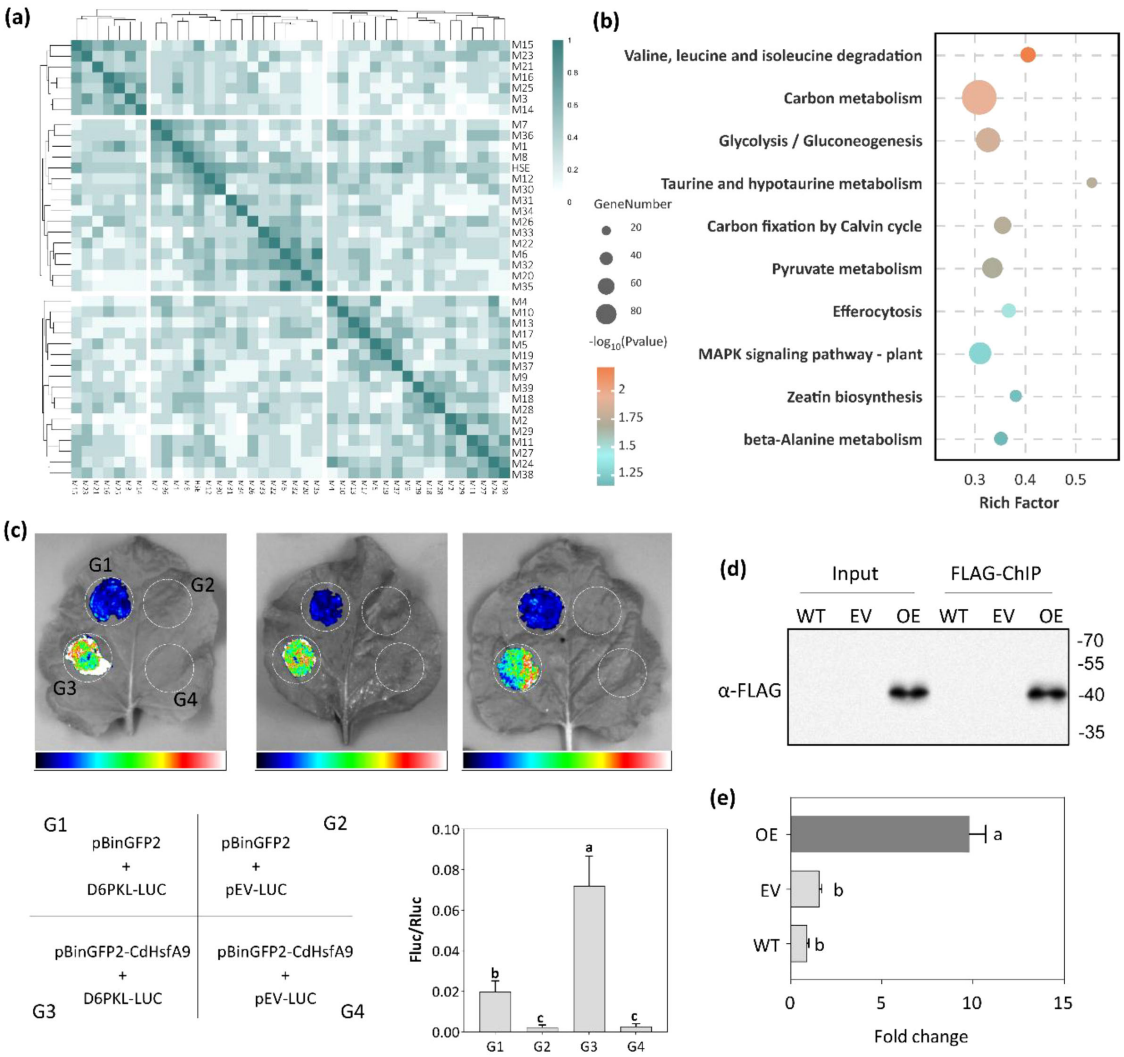
Regulatory mechanism: CdHsfA9 binding to an HSE-like motif and a *D6PKL* target. **(a)** Clustering and similarity heatmap of 39 candidate motifs identified by TF-centered yeast one-hybrid screening. The clustering was generated based on pairwise Pearson Correlation Coefficients (PCC) between the candidate motifs and the canonical Heat Shock Element (HSE) motif. **(b)** KEGG enrichment of putative CdHsfA9 target genes. **(c)** Dual-luciferase assay comparing FLuc/RLuc activity in tobacco leaves co-expressing pBinGFP2-*CdHsfA9* with pGreenII0800-LUC-*D6PKL* versus controls. **(d)** Western blot analysis of CdHsfA9-FLAG in Input and ChIP eluates from wild type (WT), empty vector line (EV), and *CdHsfA9*-OE lines. **(e)** ChIP-qPCR enrichment at the promoter of *AT5G03640* (*Arabidopsis D6PKL* homolog). Data are mean ± SD (n = 3).

To explore the regulatory mechanism underlying CdHsfA9-mediated cold tolerance, a TF-Y1H assay was conducted using the bait of pGADT7-*CdHsfA9*. The sequencing of positive clones identified 39 *CdHsfA9*-interacting motifs. To identify HSE-like targets, a motif similarity clustering analysis of these motifs and the canonical HSE was performed by PCC method. As shown in the heatmap, several candidates, like motif-21 and motif-34, clustered closely with the reference HSE motif, indicating high structural similarity (**Figure 4a**). Genome-wide promoter scanning of bermudagrass predicted putative CdHsfA9 targets. KEGG pathway analysis revealed that these target genes were enriched in metabolic pathways (including carbon metabolism, glycolysis/gluconeogenesis, and pyruvate metabolism) and signal transduction pathways such as MAPK signaling pathway and zeatin biosynthesis (**Figure 4b**). These data suggest that CdHsfA9 not only regulates the classic temperature-response modules but also is implicated in energy metabolism reprogramming and kinase-mediated signaling networks. Through alignment with public databases, motif-34 (GGTACTA) was identified as a high-confidence HSF-binding consensus. Then, based on the strict criteria of sequence similarity and spatial overlap of targeted promoters with canonical HSEs, the gene encoding serine/threonine-protein kinase D6PK-like (*D6PKL*) was prioritized as a primary candidate. The *D6PKL* promoter was found to contain motif-34 binding site, consistent with the kinase-related pathway enrichment.

To determine the binding ability of CdHsfA9 and motif-34 in the promoter of *D6PKL* in plants, a dual-luciferase assay in *Nicotiana benthamiana* leaves was performed. The results of three repeated experiments all showed that co-expression of *pGreenII_0800_LUC*-*D6PKL* with *pBinGFP2*-*CdHsfA9* led to a significant increase in the Fluc/Rluc ratio compared with control combinations (**Figure 4c**), indicating promoter activation of *D6PKL* by *CdHsfA9*. To further validate in planta binding, ChIP-qPCR was performed on FLAG-tagged OE lines using FLAG antibodies. ChIP-western blotting confirmed the specific presence of the CdHsfA9-FLAG fusion protein in the Input and ChIP eluates of the OE lines, while no signal was detected in the WT and EV negative controls (**Figure 4d**). Subsequent ChIP-qPCR analysis showed a ~10-fold enrichment of the promoter of *AT5G03640* (the *Arabidopsis* homolog of *D6PKL*) in the OE lines relative to the WT and EV controls (**Figure 4e**), supporting direct binding of CdHsfA9 to the *D6PKL* promoter region.

## 4 Discussion

The heat shock factor (Hsf) family is well established as a core regulator of thermotolerance (Guo et al., 2008; Jiang et al., 2021; Li et al., 2014), and HsfA9 has been predominantly studied in the context of seed development (Prieto-Dapena et al., 2017; Prieto-Dapena et al., 2008; Wang et al., 2024; Zinsmeister et al., 2020). By contrast, its specific functions and regulatory networks under low-temperature stress remain less defined. Here, this study examined whether and how *HsfA9* contributes to cold tolerance in a warm-season grass, hypothesizing that CdHsfA9 rewires cold responses through hormone and protein-kinase signaling.

In this study, A9-type Hsf was first identified in a warm-season bermudagrass species. First, our data position CdHsfA9 within the canonical HSF framework while highlighting lineage-specific features. CdHsfA9 contains the conserved HSF domains, exhibits nuclear localization, and activates HSE-like elements (**Figure 1b**), consistent with the nuclear site - HSE-dependent - resilience network paradigm (Carranco et al., 2010; Giesguth et al., 2015; Li et al., 2024). Meanwhile, *CdHsfA9* is rapidly induced by low temperature (**Figure 2b**), similar to the low-temperature/oxidation signals of certain rice HSFs (Mittal et al., 2009). Notably, in bermudagrass *CdHsfA9* shows preferential expression in mature leaves (**Figure 2a**), differing from the seed-specific expression typically reported for HsfA9 in species such as *Arabidopsis* and sunflower (Carranco et al., 2010; Prieto-Dapena et al., 2017; Wang et al., 2024). Together with the phylogenetic placement of CdHsfA9 alongside AtHsfA8 rather than AtHsfA9 (**Figure 1a**), these observations support these observations support functional divergence whereby monocot HsfA9-like factors act in vegetative tissues under stress, in contrast to the classical seed-associated A9 clade in dicots (Giesguth et al., 2015). In addition, heterologous overexpression of *CdHsfA9* could enhance the cold resistance of *Arabidopsis* (**Figure 3**), indicating that in monocot turfgrass, the function of the HsfA9 subfamily may have shifted from seed development to the stress adaptation of nutritional organs, representing an important functional evolution.

Second, our findings extend the HSF regulatory network by implicating auxin signaling and a serine/threonine protein kinase as critical downstream nodes. Overexpression of *CdHsfA9* significantly increased IAA content in vegetative tissues (**Figure 3b**), unlike the *hsfA8* mutant with lower IAA levels and compromised cold tolerance. This distinction is notable given that *AtHsfA8* gene is established as a key regulator of oxidative stress response (Giesguth et al., 2015). Furthermore, while previous studies have suggested an association between *HaHsfA9* and auxin signaling pathways in sunflower seed (Carranco et al., 2010), our data underscore the physiological importance of this interaction in vegetative organs, where the maintenance of auxin homeostasis is pivotal for cold adaptation. Low temperature typically suppresses auxin transport and signaling, resulting in growth inhibition and tissue damage (Rahman, 2013). Emerging evidence suggests that auxin homeostasis is linked to Hsf-mediated stress adaptation and redox homeostasis. For example, the IAA17.1/HSFA5a module enhances stress tolerance in *Populus tomentosa* by regulating flavonol biosynthesis and improving ROS scavenging (Song et al., 2024). Consistent with this model, the elevated IAA level in *CdHsfA9*-OE plants coincided with reduced MDA accumulation and enhanced antioxidant enzyme activities (**Figure 3e**, **Supplementary Figure 1**). Consequently, this places *CdHsfA9* at a potential intersection between low-temperature signaling and auxin-mediated cold tolerance, thereby mitigating oxidative damage.

Mechanistically, this regulatory module is anchored by the identification of a direct downstream target of CdHsfA9, the serine/threonine protein kinase D6PK-like (*D6PKL*), confirmed via motif-guided predictions, dual-luciferase activation, and ChIP-qPCR (**Figure 4**). Previous studies have indicated that D6PK family kinases facilitate the phosphorylation-dependent regulation of PIN-FORMED auxin transporters (Barbosa et al., 2018; Zhou and Luo, 2018). While HSFs are classically viewed as transcriptional activators of HSPs (Li et al., 2024), the engagement of *D6PKL* indicates that *CdHsfA9* also connects to kinase-centered signaling networks. Therefore, the enrichment of predicted targets in pathways related to carbon metabolism, MAPK signaling, and zeatin biosynthesis (**Figure 4b**) supports a comprehensive model wherein CdHsfA9 coordinates energy metabolism reprogramming and signal transduction alongside hormone-responsive pathways to confer cold tolerance.

In this study, a framework in which *CdHsfA9* integrates an HSE-like transcriptional module with auxin- and kinase-mediated pathways to promote cold tolerance when expressed in *Arabidopsis*. This model is in line with the observed phylogenetic proximity to *AtHsfA8* and the vegetative expression pattern, providing a mechanistic basis for the *CdHsfA9*-*HSE*-*D6PKL-IAA* axis within a broader stress-response network. However, several limitations of this study warrant further investigation. Future research should employ CRISPR-Cas9-based gene editing to generate *cdhsfA9* knockout or knockdown mutants in bermudagrass itself, which would provide definitive genetic evidence for its endogenous role. Secondly, the precise function of *D6PKL* downstream of *CdHsfA9* remains unknown, including the identity of phosphorylation substrates. Finally, the interface between CdHsfA9 activity and auxin biosynthesis or signaling requires dissection. It would be crucial to determine whether *CdHsfA9* directly regulates genes in auxin metabolism or if this is an indirect effect mediated through *D6PKL* or other intermediaries. Addressing these questions will refine our mechanistic model and pave the way for engineering multi-pathway coordinated stress resilience in crops.

## Data Availability

The datasets presented in this study can be found in online repositories. The names of the repository/repositories and accession number(s) can be found in the article/**Supplementary Material**.
