# Supplementary material for: Expanding the role of HsfA9 in cold adaptation: the bermudagrass CdHsfA9 confers cold tolerance in Arabidopsis via a novel regulatory module
Source: Front Plant Sci. 2025 Dec 19;16:1731555. doi: 10.3389/fpls.2025.1731555 (PMC12757282; doi:10.3389/fpls.2025.1731555)
Supplement: Supplementary file 1 [file DataSheet1.docx]

Supplementary Material

# Supplementary Figures


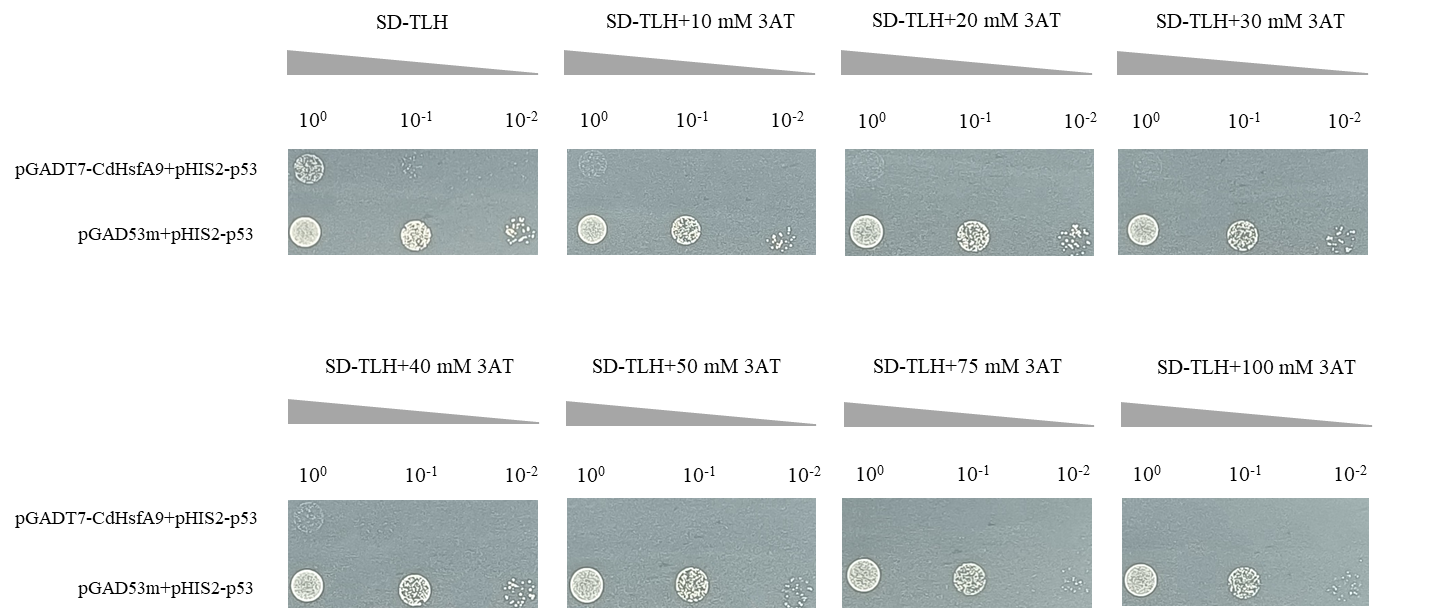
**Supplementary Figure 1.** Self-activation detection result.


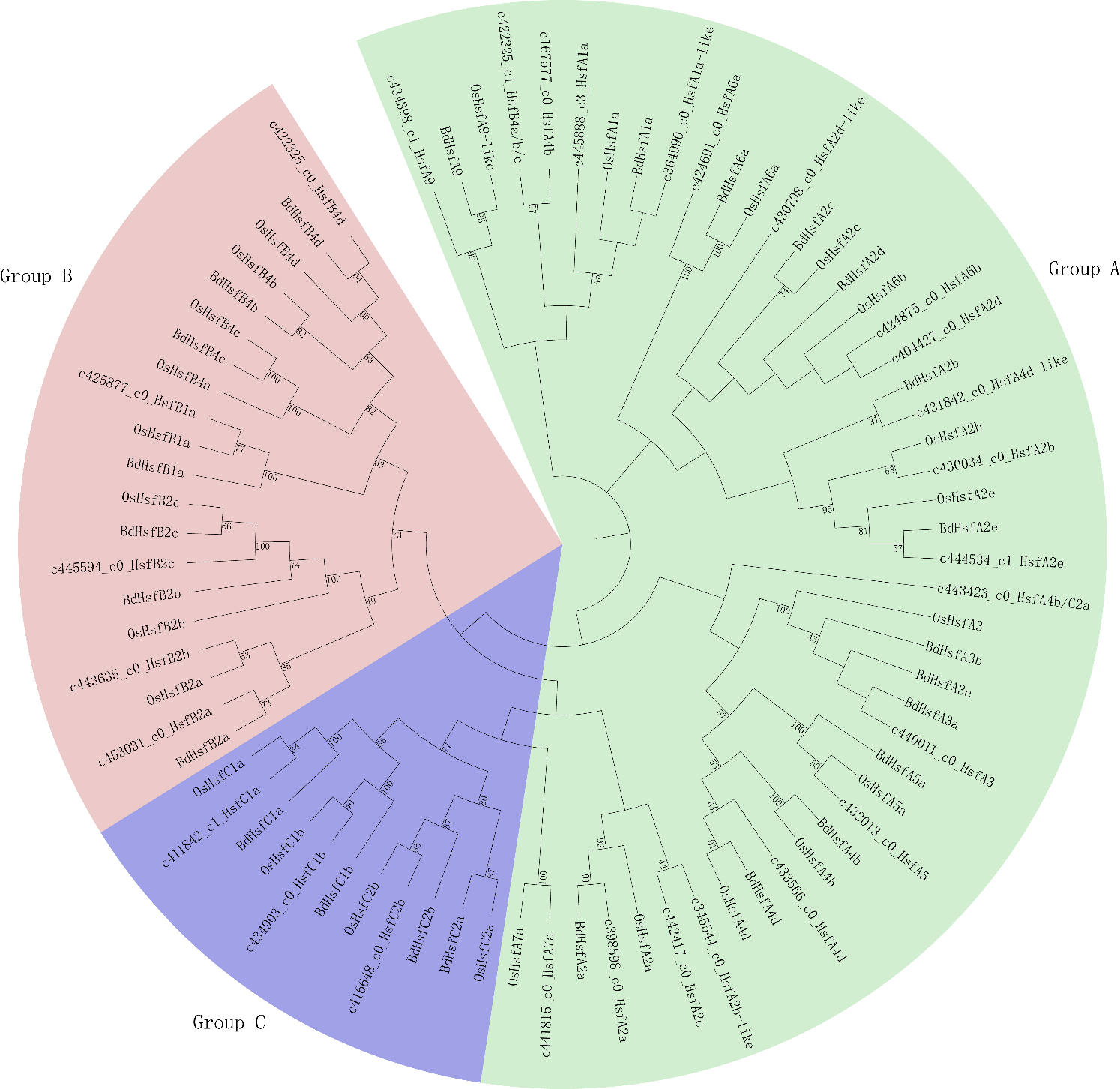


**Supplementary Figure 2.** Phylogenetic analysis of CdHsfA9 within the Hsf superfamily. The phylogenetic tree was constructed using the Neighbor-Joining method in MEGA. It includes CdHsfA9 and representative Heat Shock Transcription Factors (Hsfs) from Classes A, B, and C identified in the *Cynodon dactylon* transcriptome, as well as reference sequences from *Oryza sativa* (Os) and *Brachypodium distachyon* (Bd). The analysis confirms the classification of CdHsfA9 within the HsfA subfamily.


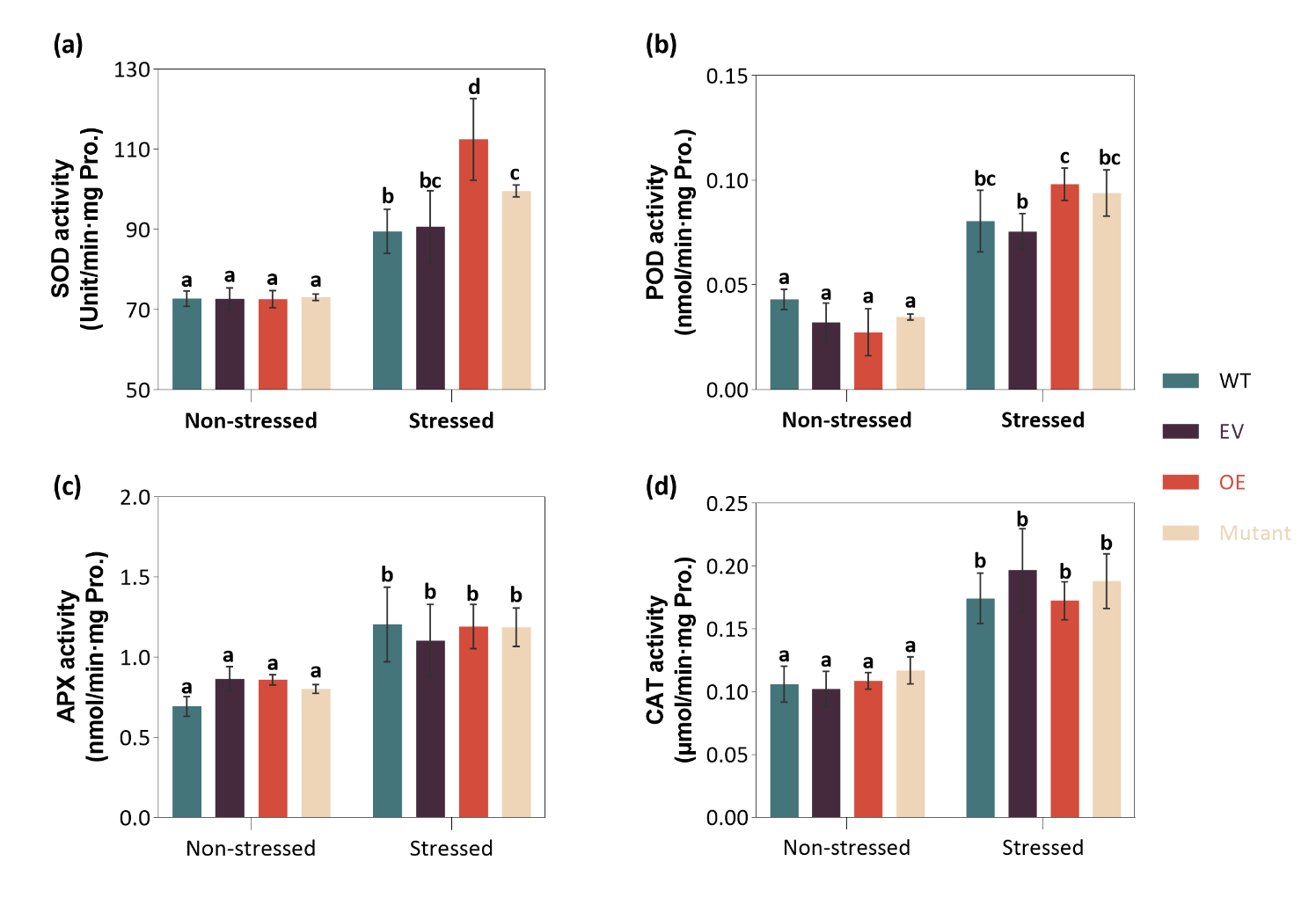


**Supplementary Figure 3.** Analysis of antioxidant enzyme activities in transgenic *Arabidopsis* under cold stress. Activities of **(a)** Superoxide dismutase (SOD), **(b)** Peroxidase (POD), **(c)** Ascorbate peroxidase (APX), and **(d)** Catalase (CAT) were measured in Wild Type (WT), Empty Vector (EV), *CdHsfA9*-Overexpression (OE), and *AtHsfA8* mutant plants under non-stressed conditions and cold stress at 5°C. Data are presented as mean ± SD (n = 3 biological replicates). Different letters indicate significant differences at P < 0.05 according to Fisher’s LSD test.

# Supplementary Tables

**Supplementary Table 1.** Primers in the study.

| Primer Name | Primer Sequence (5'-3') | Amplication |
| --- | --- | --- |
| CdHsfA9-F | ATGGGCTCGAAGAAGCGGT | ORF amplication |
| CdHsfA9-R | ACCGCTTCTTCGAGCCCAT |  |
| HsfA9-subF | cgagctcaagcttcgaattcATGGGCTCGAAGAAGCGGT | Subcellular localization |
| HsfA9-subR | cagcggcagcagccggatccTGCTTCAGTAATGACATCAGCA |  |
| HsfA9-F-AD | GGATCCCGATGGGCTCGAAGAAGCGGT | Y1H-pGADT7 |
| HsfA9-R-AD | AGCTCGAGACCGCTTCTTCGAGCCCAT |  |
| pBinGFP2-CdHsfA9-F | CTCGGCATGGACGAGCTGTACAAGGGTACCATGGGCTCGAAGAAGCGGTCCCCGCA | Dual-luciferase |
| pBinGFP2-CdHsfA9-R | TTTGCGGACTCTAGTTCATCTAGAGGATCCTCATGCTTCAGTAATGACATCAGCA |  |
| pLUC-promoter-F | GGTACCCAGGGGGCGCGTCTCGTGGC |  |
| pLUC-promoter-R | GGATCCGCGAAAGAAAATGGGGGGCG |  |
| HsfA9-oeF | tgtgtgtgcagcccgggatccATGGGCTCGAAGAAGCGG | Overexpression |
| HsfA9-oeR | tccatggtacctgcaggatccTGCTTCAGTAATGACATCAGCATATT |  |
| ChIP-qPCR-F | AACGTTACTATTTAGAAGACC | ChIP-qPCR |
| ChIP-qPCR-R | CTTAAAGATAAGCTGATT |  |
| CdActin-F | CCAAGGCAAACAGGGAGAAGA | qRT-PCR |
| CdActin-R | AGTCGGATGATAGCGTGAGGG |  |
| HsfA9-qF | GTTGTCCAAAGTCCAGACTTC |  |
| HsfA9-qR | CATGGAAATAGCAACTGC |  |
